# 

**Published:** 1885-07

**Authors:** 


JULY, 1885.

### THE AMERICAN JOURNAL OF DENTAL SCIENCE.

EDITED BY

F. J. S. GORGAS, M. D., D. D. S.

VOL. 19. THIRD SERIES. No 3

BALTIMORE:

SNOWDEN & COWMAN.

LONDON:
TRUBNER & CO., 60 PATERNOSTER ROW.

1883.

PUBLISHED MONTHLY.

$2.50 PER ANNUM,

